# Novel Ti-Ta-Hf-Zr alloys with promising mechanical properties for prospective stent applications

**DOI:** 10.1038/srep37901

**Published:** 2016-11-29

**Authors:** Jixing Lin, Sertan Ozan, Yuncang Li, Dehai Ping, Xian Tong, Guangyu Li, Cuie Wen

**Affiliations:** 1 College of Materials Science and Engineering, Jilin University, Jilin 130025, China; 2 Advanced Material Research and Development Center, Zhejiang Industry & Trade Vocational College, Zhejiang 325003, China; 3 Department of Mechanical Engineering, Bozok University, Yozgat 66100, Turkey; 4 School of Engineering, RMIT University, Victoria 3083, Australia; 5 National Institute for Materials Science, Tsukuba 3050047, Japan

## Abstract

Titanium alloys are receiving increasing research interest for the development of metallic stent materials due to their excellent biocompatibility, corrosion resistance, non-magnetism and radiopacity. In this study, a new series of Ti-Ta-Hf-Zr (TTHZ) alloys including Ti-37Ta-26Hf-13Zr, Ti-40Ta-22Hf-11.7Zr and Ti-45Ta-18.4Hf-10Zr (wt.%) were designed using the d-electron theory combined with electron to atom ratio (e/a) and molybdenum equivalence (Mo_eq_) approaches. The microstructure of the TTHZ alloys were investigated using optical microscopy, XRD, SEM and TEM and the mechanical properties were tested using a Vickers micro-indenter, compression and tensile testing machines. The cytocompatibility of the alloys was assessed using osteoblast-like cells *in vitro*. The as-cast TTHZ alloys consisted of primarily β and ω nanoparticles and their tensile strength, yield strength, Young’s modulus and elastic admissible strain were measured as being between 1000.7–1172.8 MPa, 1000.7–1132.2 MPa, 71.7–79.1 GPa and 1.32–1.58%, respectively. The compressive yield strength of the as-cast alloys ranged from 1137.0 to 1158.0 MPa. The TTHZ alloys exhibited excellent cytocompatibility as indicated by their high cell viability ratios, which were close to that of CP-Ti. The TTHZ alloys can be anticipated to be promising metallic stent materials by virtue of the unique combination of extraordinarily high elastic admissible strain, high mechanical strength and excellent biocompatibility.

With the development of society and the continuous improvement of standards of living, people are facing increasing social pressure, which is accompanied by increased rates of occurrence cardiovascular diseases (CVDs). According to statistical data published by the World Health Organization (WHO) in 2012, the number of the people dying from CVDs is expected to increase from 17 million in 2008 to 25 million in 2030^1^. With the wide application and rapid development of micro-traumatic intervention treatment, the implantation of vascular stents is acknowledged to be one of the most effective approaches for CVDs^2^,^3^,^4^.

At present, the most commonly used stent materials include metal and polymer materials. Compared with polymer materials, metallic stents exhibit stable performance and therefore can provide preferable supporting strength. Nitinol (Ni-Ti), stainless steel (316L SS), cobalt-chromium (Co-Cr) alloy, tantalum (Ta), pure iron (Fe), platinum-iridium (Pt-Ir) alloy, and magnesium (Mg) alloys are the metallic biomaterials used for manufacturing stents^5^. Among these metallic stent materials, pure Fe and Mg alloys are the two metals that have been used for making biodegradable coronary stents^6^. 316L SS, which is used as a balloon-expandable stent material, is well-known for its low yield strength (331 MPa) and high Young’s modulus (190 GPa)^7^,^8^, whilst Ni-Ti alloy with Young’s modulus in the range of 75–83 GPa and yield strength ranging from 195 to 690 MPa^7^,^9^ is widely used as a self-expandable stent material. However, both of them contain Ni, which may trigger a local immune response and inflammatory reactions^10^,^11^,^12^. In addition, 316L SS and Co-Cr alloys cause concerns due to its inadequate MRI (magnetic resonance imaging) compatibility. Ta possesses excellent corrosion resistance but has poor mechanical properties; furthermore, the biocompatibility and haemocompatibility of Pt-Ir alloy need to be verified further^6^. It is well known that implant materials that have a high magnetic susceptibility are not appropriate for magnetic resonance imaging diagnostic in surgeries. In this regard, it is important to notify that Co-Cr alloys have a relatively high magnetic susceptibility, being ~1370 × 10^−6^ cm^3^/g^13^ in comparison to Ti with a magnetic susceptibility of 180 × 10^−6^ cm^3^/g^14^. On the other hand, Ti and its alloys are preferred candidates for metallic stents due to their excellent biocompatibility and corrosion resistance^15^. Biehl *et al.*^16^ demonstrated that the excellent haemocompatibility together with good mechanical properties of Ti-Ta and Ti-Nb alloys make them promising stent materials. In particular, apart from not inducing biological toxicity which can not be avoided for conventional stent materials, some new β-type Ti alloys display excellent hyperelasticity, good corrosion resistance, favorable biocompatibility and fracture toughness similar to that of α + β type Ti alloys^17^,^18^,^19^. This type of Ti alloys has become the hotpots in the field of metallic biomaterials due to their superior biocompatibility and biocompatible mechanical properties. In the design of new β-type Ti alloys, alloying elements can be divided into alpha (α) stabilizers, beta (β) stabilizers and neutral elements according to their influences on the β transition temperature of Ti and their solubilities in α and β phases. Furthermore, β stabilizers are divided into isomorphous and eutectoid types. Considering the biocompatibility and the stability of β phase, β stabilizers such as Mo, Ta, Nb, Hf, Pd and Fe are usually selected as primary additive elements in the design of a β-type biomedical Ti alloy. Moreover, Zr can be used as a β stabilizer when used with combination of other β stabilizers such as Ta and Nb, and improve the performance of the Ti alloys^20^,^21^,^22^,^23^,^24^,^25^. In this study, Ta, Zr and Hf were selected as the β stabilizers in order to achieve improved performance of the Ti alloys. Meanwhile, based on the d-electron alloy design method^26^, electron to atom ratio ((e/a)_ratio_)^27^ and the molybdenum equivalence (Mo_eq_) requirements^28^, three new β-type Ti alloys were designed, aiming at an unique combination of mechanical properties of high elastic admissible strain and high mechanical strength.

Hafnium (Hf) is a β stabilizer but there are only a few studies reported the influences of Hf in Ti alloys^29^,^30^, to date. Therefore, further research on the effect of Hf on the biocompatibility and mechanical properties of titanium alloys is highly pertinent. In this study, we select three β stabilizers, that is, Ta, Hf, and Zr, as the Ti alloying elements in order to develop new β Ti alloys for metallic stent materials. All of the three alloying elements are non-ferromagnetic metals^31^, which is of great importance for stent applications.

## Results and discussion

### Microstructural characterization

**Figure 1:**
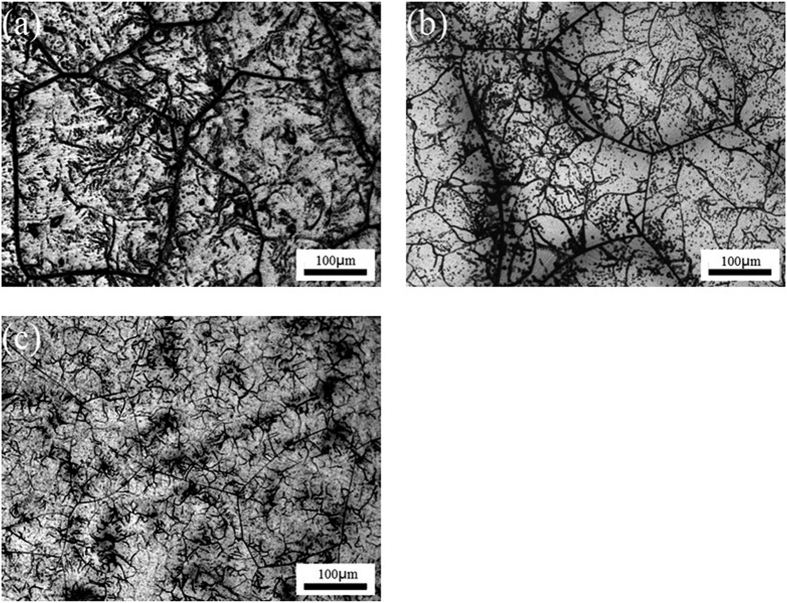
Optical micrographs of as-cast TTHZ alloys: (**a**) TTHZ-1 (Ti-37Ta-26Hf-13Zr), (**b**) TTHZ-2 (Ti-40Ta-22Hf-11.7Zr), and (**c**) TTHZ-3 (Ti-45Ta-18.4Hf-10Zr).

**Figure 2:**
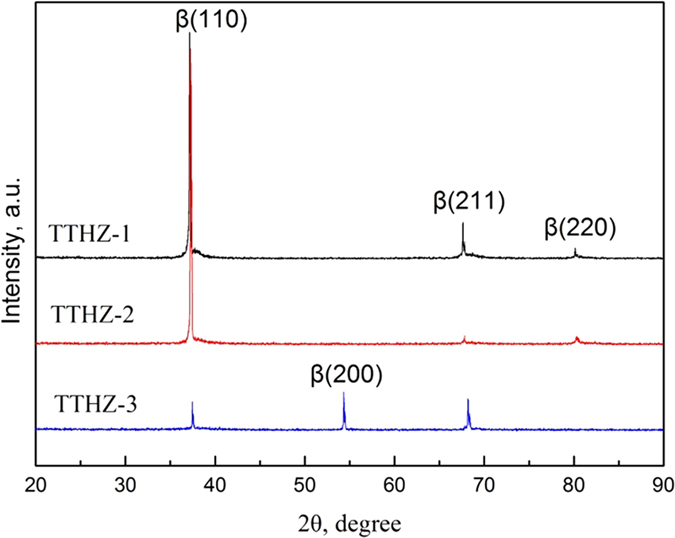
XRD patterns of as-cast TTHZ alloys: (TTHZ-1: Ti-37Ta-26Hf-13Zr, TTHZ-2: Ti-40Ta-22Hf-11.7Zr, TTHZ-3: Ti-45Ta-18.4Hf-10Zr).

Figure 1 shows the microstructures of the as-cast TTHZ alloys. It can be seen that the grain boundary was straight and clear, and that the polygonal grains were of various sizes, the average grain size of TTHZ-1, TTHZ-2, and TTHZ-3 is 354.6 μm, 335.3 μm, 341.2 μm, respectively. The density is 8.7, 8.6 and 8.7 g/cm^3^ for TTHZ-1, TTHZ-2, and TTHZ-3, respectively, which is slightly higher than that of 316L SS stainless steel (7.9 g/cm^3^); representing an intermediate range among the metallic stent materials because it is lower than those of tantalum (16.6 g/cm^3^) and Co-Cr alloy (~9.2 g/cm^3^), but higher than that of Nitinol (~6.7 g/cm^3^). Figure 2 shows the XRD patterns of the TTHZ alloys, from which it can be seen that the as-cast alloys only contained a β phase. TTHZ-1 and TTHZ-2 alloys exhibited similar XRD patterns. TTHZ-3 showed a significantly weakened (110)_β_ peak, accompanied by the appearance of the (200)_β_ peak and the disappearance of the (220)_β_ peak, compared to TTHZ-1 and TTHZ-2.

**Figure 3:**
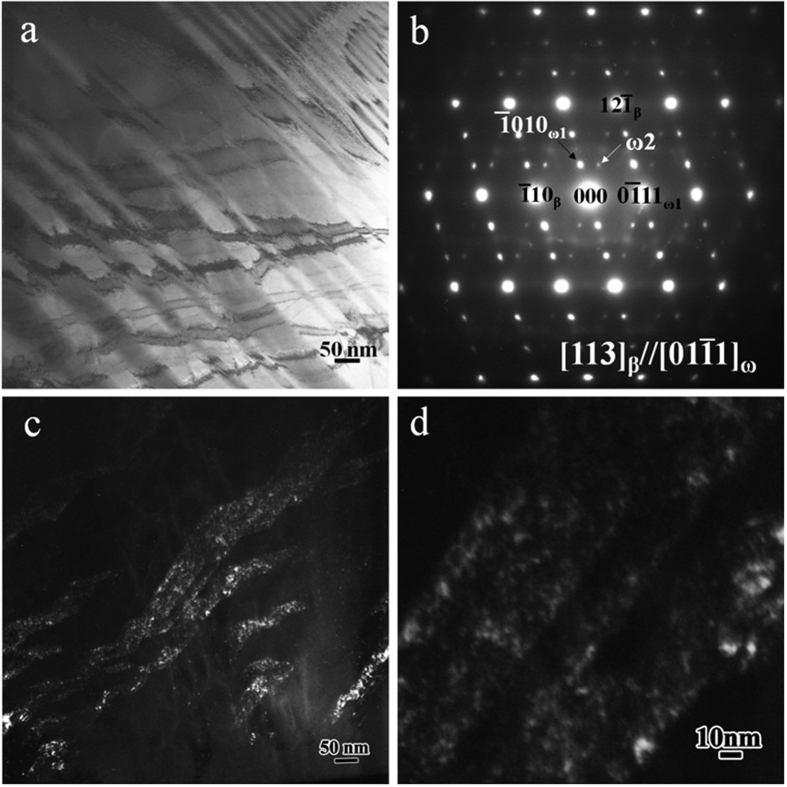
TEM images of TTHZ-3 (Ti-45Ta-18.4Hf-10Zr): (**a**) bright field image, (**b**) the corresponding selected area electron diffraction of (**a**). (**c**) The dark field image obtained using the (-1010)_ω1_ diffraction spot. (**d**) Enlarged image of a local region in (**c**).

Detailed microstructure was investigated using TEM observation. The microstructure of the TTHZ alloy samples was mainly a β matrix phase embedded with ω nanoparticles. The particle size of ω phases were estimated to be approximately 5 nm in TTHZ-1, and 2 nm in TTHZ-2 and TTHZ-3 based on TEM dark field analysis. The bright field image, the diffraction pattern in selected areas, and the TEM image of the ω phase under dark field conditions, of the TTHZ-3 alloy are shown in Fig. 3. The diffraction spots in the [113] zone axis of β is shown in Fig. 3b. It can be seen that the diffraction spots were composed of three sets of diffraction patterns: one from the 113 zone axis of β-Ti, and the other two from the variants 1 and 2 of ω. However, no peaks of ω phases appeared in the XRD pattern (Fig. 2), which is probably due to the small size of the ω phases. It can be seen that the size of the ω particles was about 2 nm. The distribution of the ω particles was not uniform in the matrix (Fig. 3c). This is probably caused by the different variants of the ω phases. Similar results were reported for β-type Ti-30Zr-5Mo, Ti-24Nb-2Zr, and Ti-19Zr-10Nb-1Fe elsewhere^32^,^33^,^34^.

### Micro-hardness

**Figure 4:**
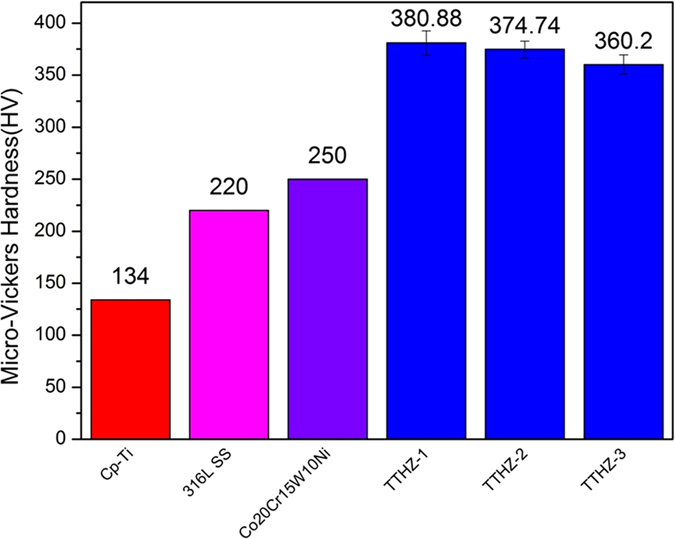
Micro-hardness of TTHZ alloys in comparison with the commonly used metallic stent materials published in the literature [35,36,37]. (TTHZ-1: Ti-37Ta-26Hf-13Zr, TTHZ-2: Ti-40Ta-22Hf-11.7Zr, TTHZ-3: Ti-45Ta-18.4Hf-10Zr).

The micro-hardness of the TTHZ alloys is shown in Fig. 4. The average micro-hardness was 380.9 ± 8.0 HV, 374.7 ± 5.8 HV, and 360.2 ± 6.4 HV for TTHZ-1, TTHZ-2, and TTHZ-3, respectively; compared to 134.0 HV for CP-Ti^35^, 220.0 HV for 316 L SS^36^, and 250.0 HV for Co20Cr15W10Ni^37^. There was no significant difference (ρ > 0.05) between the micro-hardness of TTHZ-1 and TTHZ-2, but both of them were significantly higher than that of TTHZ-3. It can be seen that the TTHZ alloys, in their as-cast state, exhibited higher hardness values in comparison to the conventional stent materials. The micro-hardness value of TTHZ-1 is around 2.84 times that of CP-Ti and 1.73 times that of 316L SS. These results indicate that the TTHZ alloys can favorably bear a higher compression load in the blood vessels compared to conventional stent materials. Alloying elements including Ta, Hf, and Zr were added into the TTHZ alloys, and they played a solid-solution strengthening role. In addition, since the nanoparticles of the ω phase is exceptionally hard, a small amount of ω phase can significantly enhance the micro-hardness of the TTHZ alloys. The hardness value of TTHZ alloys can be arranged, in descending order, as: TTHZ-1, TTHZ-2, and TTHZ-3. This can be attributed to the solid-solution strengthening effect that is determined by the atomic radius difference of the alloying elements and the matrix. In this study, the atomic radius is 1.76 Å, 2.16 Å, 2.16 Å and 2.09 Å for Ti, Hf, Zr, and Ta, respectively. The greater the radius differences between the atoms of the matrix (Ti) and the alloying element, the better the solid-solution strengthening effect. Therefore, with increasing Ta content, the solid-solution strengthening effect of these alloys decreased, which then gave rise to the reduced hardness of the higher Ta containing alloy (TTHZ-3).

Shear bands, which were seen around the micro-hardness indents of the TTHZ alloys, present a consecutive wavy form as seen in Fig. S1. The observation of shear bands is a case often encountered around the hardness indents of metallic glass materials having an amorphous structure^38^,^39^,^40^. However, observation of the shear bands in a crystalline material is scarcely reported^41^,^42^. Shear bands formed around hardness indents were observed in β titanium alloys because the alloys showed no strain hardening after yielding^41^. The shear bands were emanated from the edges of the micro-Vickers indents (Fig. S1). No cracks were detected around the micro-Vickers indents of the TTHZ alloys. By considering the principle that the volume of materials is constant during the plastic deformation, deformations in the elastic-perfect plastic materials occur through piling up mechanism^43^. The white arrows indicate the piling up mechanism peculiar to shear band formation (Fig. S1).

### Compressive properties

**Figure 5:**
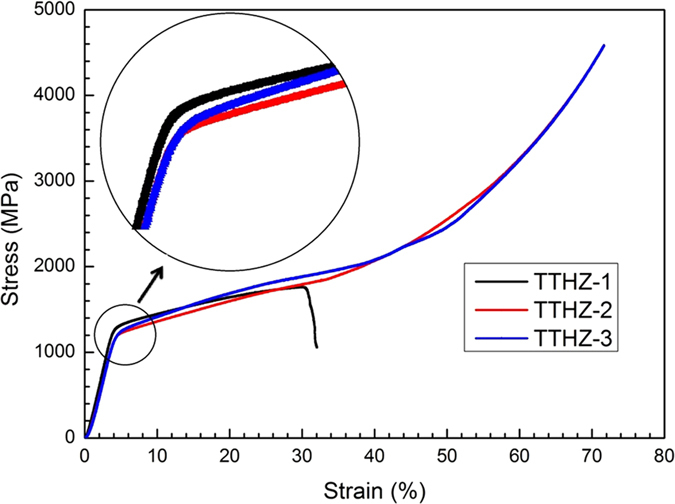
Compressive stress-strain curves of the as-cast TTHZ alloys. (TTHZ-1: Ti-37Ta-26Hf-13Zr, TTHZ-2: Ti-40Ta-22Hf-11.7Zr, TTHZ-3: Ti-45Ta-18.4Hf-10Zr).

The compressive stress-strain curves of the TTHZ alloys are given in Fig. 5. The compressive yield strength was 1158.0 ± 49.4 MPa, 1154.0 ± 31.2 Mpa and 1137.0 ± 26.5 MPa for TTHZ-1, TTHZ-2 and TTHZ-3, respectively. Among the compressive yield strength of the three TTHZ alloys, no significant (ρ > 0.05) difference was observed. The sequence of the compressive yield strength is accorded with the sequence of their micro-hardness values. The images of the TTHZ samples after compression test are shown in Fig. S2. A fracture in the TTHZ-1 alloy sample has been observed (Fig. S2 (a,a’)). The compressive plastic strain of TTHZ-1 was measured as 34.0 ± 2.8%. A careful examination revealed that the TTHZ-1 alloy sample underwent fracture at an angle of 45° along the specimen longitudinal axis. On the other hand, TTHZ-2 and TTHZ-3 samples did not undergo fracture during compression test (Fig. S2 (b) and (c)). This is consistent with the stress-strain curves shown in Fig. 5. Shear band formation is crucial for increasing plastic strain^43^. Schroers *et al.*^44^ indicated that an enhanced ductility of a bulk metallic glass under compression can be explained in terms of multiple shear band formations which prevent crack initiation. Consequently, the ductility of TTHZ alloys under compression may be resulted from multiple shear band formations. The high compressive yield strength of the TTHZ alloys may be stemmed from the joint effect of the solid-solution strengthening of Ta, Hf, and Zr on the β-Ti matrix and the precipitation of the ω nanoparticles. It can be seen that the compressive strength and the strain of TTHZ-2 and TTHZ-3 are highly desired mechanical properties for metallic stent materials.

### Tensile properties of the TTHZ alloys

Figure S3 shows the tensile stress-strain curves of the TTHZ alloys. The comparison of the tensile properties including the tensile strength, yield strength, Young’s modulus, and elastic admissible strain of the TTHZ alloys with conventional metallic stent materials are listed in Table 1. As can be seen, the tensile strengths of TTHZ-1, TTHZ-2, and TTHZ-3 were 1000.7 ± 108.2 MPa, 1160.2 ± 28.3 MPa, and 1172.8 ± 45.1 MPa, respectively. When a ranking in terms of tensile strength is made, TTHZ alloys are ranked TTHZ-3 > TTHZ-2 > TTHZ-1. It can be seen that the tensile strength of TTHZ-1 was significantly (ρ < 0.05) lower than that of TTHZ-2 and TTHZ-3; however, there was no significant difference (*ρ* > 0.05) in the tensile strengths between TTHZ-2 and TTHZ-3. By comparison with the conventional metallic stent materials, including 316L SS^7^,^8^, tantalum^7^, CP-Ti^7^,^8^, Nitinol^7^,^9^ and Co-Cr^7^,^8^,^9^, it can be found that the tensile strength of the TTHZ alloys was close to the highest tensile strength (951.0–1220.0 MPa) of Co-Cr, which has the maximum tensile strength among the current existing metallic stent materials. Meanwhile, the tensile yield strengths of TTHZ alloys were 1000.7 ± 108.2 MPa, 1132.2 ± 29.4 MPa, and 1114.3 ± 61.0 MPa, respectively. Ranking of yield strength of TTHZ alloys is TTHZ-2 > TTHZ-3 > TTHZ-1. It can be seen that the yield strength of TTHZ-1 was significantly (ρ < 0.05) lower than that of TTHZ-2; which showed no significant difference (ρ > 0.05) from that of TTHZ-3. It can be deduced that the mechanical properties of the TTHZ alloys could enable stent design taking shape of thin struts. The strut thickness of 316L stainless steel stent is 140 μm (e.g., BX Velocity^®^/Johnson & Johnson) and of CoCr alloy (L-605) stent is 81 μm (e.g., Vision TM/Abbott)^45^. It is worth noting that high mechanical strength is a prerequisite in the design of stents with thin strut thickness^45^, and thinner struts contribute to reduction in restenosis rates^45^,^46^,^47^. The TTHZ alloys with tensile yield strength in the range of 1000.7–1132.2 MPa are promising candidate materials for minimizing strut thickness of stents, which thereby could lead to a reduced restenosis rate. By taking the yield strength into consideration, being about 340 MPa, of 316L stainless steel (BX Velocity^®^/ Johnson & Johnson)^45^, one could readily estimate that the strut thickness of 140 μm could be decreased if a stent material with a yield strength greater than 340 MPa is used. TTHZ-2 alloy possessing about 2.22 times higher yield strength than that of CoCrL-605 (VisionTM/Abbott) stent material, therefore it allows stent design with further decreased strut thickness, a value less than 81 μm. The yield strength of TTHZ alloys is higher than those of the existing metallic stent materials. It is commonly known that bonding, microstructure and atomic nature are the internal factors determining the strength of a material. Microstructure is the main factor, including four strengthening mechanisms, namely solid solution strengthening, deformation strengthening, precipitation and dispersion strengthening, and grain and sub-grain strengthening. The first three mechanisms improve the strength of the material, but also reduce the ductility. In this study, the deformation strengthening does not exist in the as-cast alloy. For grain and sub-grain strengthening, the smaller grain size, the greater the unit volume of grain boundary and sub-grain boundary area, the higher strength of the alloy, however, the average grain size of the three alloys is very close since the average grain size is 354.6 μm, 335.3 μm, 341.2 μm for TTHZ-1, TTHZ-2, and TTHZ-3, respectively. Hence, the difference stemmed from the grain and sub-grain strengthening is limited. It can be deduced that the variation in the strength of the TTHZ alloys is mainly a combined effect of the solid-solution strengthening and the precipitation of the ω nanoparticles. As observed using TEM, the size of the ω particles in TTHZ-1 (~5 nm) is larger than that in TTHZ-2 (~2 nm) and TTHZ-3 (~2 nm). A small size difference of ω nanoparticles could cause a significant effect on the mechanical properties of BCC metals^48^. It has been demonstrated that when the ω particle size is around 1~3 nm, this phase does not show a noticeable effect on the mechanical properties; however, when the particles are slightly larger (that is, with a size ≥5 nm), the precipitation of the ω nanoparticles led to a significant enhancement in the micro-hardness and the strength, and also an embrittlement of the material^48^. The smaller ω nanoparticles in TTHZ-2 and 3 resulted in an increase in the micro-hardness (Fig. 5) and tensile strength (Fig. S3), compared to TTHZ-1.

The Young’s modulus of TTHZ-1, TTHZ-2 and TTHZ-3 was 79.1 ± 2.5, 71.7 ± 2.3 and 77.0 ± 3.2 GPa, respectively. Ranking of TTHZ alloys in terms of Young’s modulus is TTHZ-1 > TTHZ-3 > TTHZ-2. The Young’s modulus of TTHZ alloys was close to that of Nitinol (Austenite) (83.0 GPa). The elongation at rupture of TTHZ-1, TTHZ-2 and TTHZ-3 was measured 1.32 ± 0.18%, 2.54 ± 0.38% and 2.35 ± 0.84%, respectively. Not only the TTHZ alloys satisfy the key mechanical property requirements for use as metallic stent materials, but also possess excellent elastic strain behavior. Elastic admissible strain (δ) is defined as the ratio of yield strength over modulus (δ = σ_ys_/E)^49^,^50^. It is a useful parameter in the design of biomedical implant materials. The higher the elastic admissible strain, the more desirable the material is for such applications^49^,^50^. The elastic admissible strain of TTHZ-1, TTHZ-2, and TTHZ-3 were calculated to be 1.32 ± 0.18%, 1.58 ± 0.05%, and 1.50 ± 0.13%, respectively, which are far greater than that of Ti-6Al-4V (δ = 0.79%)^51^, 316 L SS (0.17) and Nitinol (0.23-0.83) (See Table 1). The elastic admissible strain of the TTHZ alloys are close to those of bulk metallic glasses such as Vitreloy 1 (δ = 2%)^52^ and metallic glass matrix composites such as Ti-18Zr-12V-5Cu-17Be (δ = 1.7%)^53^. Therefore, apart from being promising for applications in metallic stent materials, similar to bulk metallic glasses, the new TTHZ alloys developed in this study possess high potential to be widely used in the fields of sporting equipment such as golf club heads, electronics, medical devices and defense applications. Compared to bulk metallic glasses, the TTHZ alloys have the following advantages: (1) Large ingots of TTHZ alloys can be directly cast molded; (2) The as-cast TTHZ alloys show a certain level of plasticity; furthermore, the β phase with a b.b.c. structure of the TTHZ alloys can be subjected to thermomechanical processing after a solution treatment. In contrast, bulk metallic glasses (BMGs) such as Vitreloy 1 can only be produced at limited size which is determined by their glass forming ability. This would impede the design of biomedical devices using BMGs. Secondly, BMGs show almost zero plasticity and cannot be thermomechanically processed even after a solution treatment. Third, many BMG alloys contain toxic elements such as beryllium (Be) and nickel (Ni) which leads to long term biosafety concerns. The tensile strength, yield strength, Young’s modulus, elastic admissible strain, and elongation at rupture were measured in the ranges of 1000.7–1172.8 MPa, 1000.7–1132.2 MPa, 79.1–71.7 GPa, 1.32–1.58%, and 1.32–2.54%, respectively; these are highly desirable mechanical properties for self-expanding stent materials.

The fracture surfaces of the TTHZ alloys after tensile test are shown in Fig. S4. It can be seen that the fracture surface of TTHZ-1 exhibited a quasi-cleavage fracture: a piece of tearing edge was generated in the grain boundary, and tiny, shallow dimples were found within the grains, indicating a limited plasticity of the alloy (Fig. S4a). This is consistent with the brittle fracture nature shown in the stress-strain curve (Fig. S3a). Careful examination of the microstructure of the alloy (Fig. 1a) reveals that the grain boundaries of TTHZ-1 were thicker than those of TTHZ-2 and TTHZ-3. Thus the brittle fracture along its grains was more likely to occur in TTHZ-1. This is because brittle fractures would form nuclei and expand preferentially along disordered, high energy grain boundaries. In other words, TTHZ-1 exhibited a fracture characteristic of intergranular fracture. In contrast, no tearing edge was observed on the fracture surfaces of TTHZ-2 and TTHZ-3 (Fig. S4(b,c)). The fracture surface of TTHZ-2 was dominated by dimples with different sizes, indicating a deformation with better plasticity; whilst the fracture surface of TTHZ-3 displayed relatively shallow vein pattern, due to its lower plasticity compared to TTHZ-2. In general, the greater the dimple size (i.e., the greater the average diameter and the deeper the dimple), the better the plasticity of the material. The tensile results indicated that the TTHZ-2 possessed a certain level of plasticity, which is better than that of TTHZ-3; but TTHZ-1 showed limited plasticity.

### Cytocompatibility of the TTHZ alloys

Biocompatibility testing of new materials is an indispensable part of the development of new materials for clinical use. One effective approach to judging the cytocompatibility of materials is to observe the growth and proliferation of osteoblast cells (SaOS2) on the surface of such a material^54^. In this study, SaOS2 cells were adopted to characterize the cytocompatibility of the TTHZ alloys.

**Figure 6:**
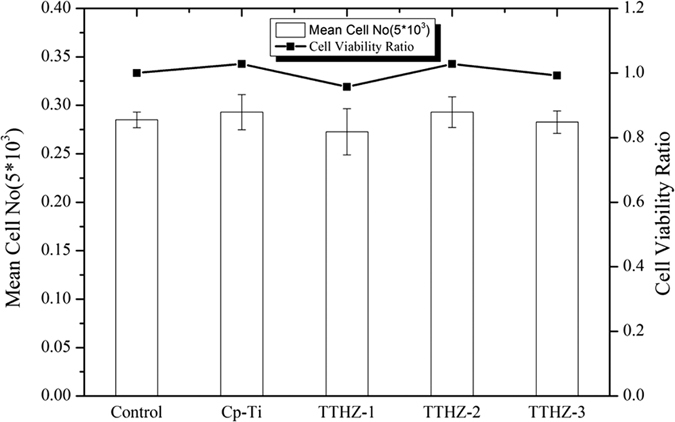
Cell viability ratio of TTHZ alloys and CP-Ti after cell culture for 7 d. (TTHZ-1: Ti-37Ta-26Hf-13Zr, TTHZ-2: Ti-40Ta-22Hf-11.7Zr, TTHZ-3: Ti-45Ta-18.4Hf-10Zr).

Figure 6 shows the cell viability ratio (CVR) values used for assessing the *in vitro* cytotoxicities of the TTHZ alloys, compared to CP-Ti. The cell viability ratio (CVR) was calculated using the equation given by^55^:




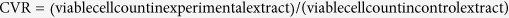




It can be seen that the CVR ratio is 1.03, 0.96, 1.03, and 0.99 for CP-Ti, TTHZ-1, TTHZ-2, and TTHZ-3, respectively. They are all similar to that of the control group; though, TTHZ-2 and CP-Ti showed slightly higher CVR ratio among the materials assessed. Hence, it can be deduced that the TTHZ alloys possess excellent cytocompatibility.

Figure S5 shows a comparison of the cell adhesion density for the TTHZ alloys and CP-Ti after 7 days of cell culture. There were no significant (ρ > 0.05) differences among the cell adhesion density (cm^−2^) of TTHZ alloys and CP-Ti. As can be seen from Fig. S5, the average cell adhesion density of the TTHZ alloys was approximately the same as that of CP-Ti. That is to say, Ta, Hf, and Zr presented similar biocompatibilty with Ti, and the variation of the Ta, Hf, and Zr contents had little influence on cell adhesion density. This is in good agreement with the results published in previous studies that Ta, Hf, and Zr are all biocompatible elements^21^,^29^.

**Figure 7:**
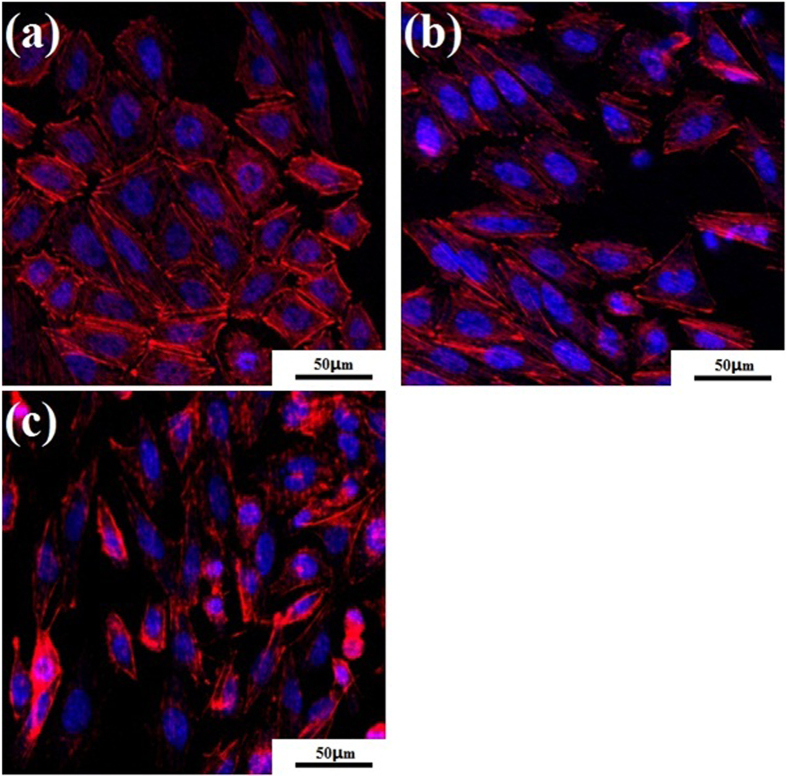
Confocal microscope images of SaOS2 cell attached on to surface of TTHZ alloys after cell culture for 7 d: (**a**) TTHZ-1 (Ti-37Ta-26Hf-13Zr), (**b**) TTHZ-2 (Ti-40Ta-22Hf-11.7Zr), and (**c**) TTHZ-3 (Ti-45Ta-18.4Hf-10Zr).

The SaOS_2_ cells on the surface of the TTHZ alloys after culture for 7 d were observed by using confocal microscope and the cell morphologies on the TTHZ alloys are shown in Fig. 7. It can be seen that extensive red actin filaments were generated on the surfaces of TTHZ alloy specimens, and the cells exhibited corner angles with oval nuclei (blue). Meanwhile, focal adhesion was found around the cells, and the cells had made contact with each other on each of the surfaces of the three alloys. The SaOS_2_ cells had spread over the surfaces, displaying healthy attachment, growth and proliferation on the TTHZ alloy surfaces.

SEM was also used to observe the morphology of the SaOS2 cells after cell culture on the TTHZ alloys for 24 h. The adhesion of the SaOS2 cells on the surfaces of the TTHZ alloys showed many filopodia in various directions around the near-oval osteoblast cells that were anchored on the surfaces of the Ti alloys. The formation of filopodia on the surfaces after cell culturing on the TTHZ alloys indicated a strong initial adhesion and healthy growth of the cells^55^. A typical image showing the morphology of the cells on TTHZ-2 is shown in Fig. S6.

## Conclusions

In order to develop new Ti alloys for metallic stents applications, a new series of Ti-Ta-Hf-Zr alloys including Ti-37Ta-26Hf-13Zr, Ti-40Ta-22Hf-11.7Zr and Ti-45Ta-18.4Hf-10Zr were designed using the d-electron alloy design method combined with electron to atom ratio (e/a) and molybdenum equivalence (Mo_eq_) approaches. The microstructures, mechanical properties and cytocompatibility of the TTHZ alloys were investigated. The following conclusions can be drawn from this study:

1. The as-cast TTHZ alloys exhibited primarily β and a small amount of ω nanoparticles, which were characterized by optical microscopy, XRD and TEM.
2. Owing to the combined effect of solid-solution strengthening of Ta, Hf, and Zr on the β-phase and the ω nanoparticles, the as-cast TTHZ alloys exhibited exceptionally high micro-hardness and compressive yield strength, which were ranged from 360.7 to 388.9 HV, 1137 to 1158 MPa, respectively. It is worth noting that such high compressive yield strength is highly desirable for metallic stent materials which can be exploited to bear the compression load in blood vessels. Shear band formation, which is an extraordinary deformation mechanism for crystalline titanium alloys, constituted the main failure mechanism during indentation and compressive loading. The TTHZ alloys exhibited a ductile deformation manner through the formation of multiple shear bands under compression.
3. The tensile strength, yield strength, and Young’s modulus of the TTHZ alloys were measured as being between 1000.67-1172.82 MPa, 1000.67-1114.33 MPa, and 71.74-79.11 GPa, respectively. The combination of high yield strength and comparatively low Young’s modulus resulted in enhanced elastic admissible strain. The TTHZ alloys exhibited unexpectedly high elastic admissible strains; in particular, the elastic admissible strain of TTHZ-2 reached 1.58%, which is much higher than that of the conventional metallic stent materials such as 316L SS, tantalum, Nitinol, and Co-Cr alloy, and is close to that of the bulk metallic glasses.
4. The TTHZ alloys exhibited excellent cytocompatibility on osteoblast-like cells (SaOS2). This study further confirmed that Hf is at the same cytocompatibility level with Ti, Zr, and Ta.
5. Overall, our results demonstrated that the TTHZ alloys possess high strength (compressive yield strength, tensile strength, and tensile yield strength), elastic admissible strain, micro-hardness, and excellent cytocompatibility. The TTHZ alloys with the highly desirable mechanical properties satisfy the mechanical requirements of self-expandable stents applications. The high yield strength combined with high elastic admissible strain provides flexible design with thinner struts which has a high potential to reduce restenosis rates in metal stents.

## Materials and Methods

### Alloy design

The d-electron alloy design method has been reported to be an effective method of designing Ti alloys^56^,^57^. In this study, we designed three Ti-Ta-Hf-Zr alloys for metallic stent applications. The average bond orders 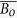 of the three alloys were 2.952, 2.942, and 2.940, respectively; while the average metal d-orbital energy levels 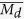 were 2.611, 2.589, and 2.573, respectively, with the corresponding positions shown in Fig. S7. TTHZ alloys were located in the β phase region and close to the β/β + ω phase boundary.

The average valence electron concentration refers to the average number of valence electrons in each atom of these alloys, and is represented by e/a. By using the ultra-soft pseudo potential method, Ikehata *et al.*^58^ explained that the critical valence electron concentration e/a should be approximately 4.20 under which the binary alloy Ti-X can exist stably with a β structure. Besides, they pointed out that the average valence electron concentration of alloys determines the stability of β-type Ti alloys to some extent. In this study, the e/a ratio of the three Ti alloys we designed are 4.205, 4.216, and 4.244, respectively (Fig. S7). The e/a ratio is slightly greater than the critical valence electron concentration (4.20), thus these alloys were expected to be β-type alloys.

Additionally, Mo equivalence can also be used to measure the stability of β-type Ti alloys^59^. When the Mo equivalence of the designed alloys is ≥8, the alloys are usually metastable β-type, or near-β-type Ti alloy^59^. In this study, the Mo equivalence of the three TTHZ alloys we designed are8.10, 8.80, and 9.90, respectively (Fig. S7), which are within the ideal range for a stable β phase of Ti alloys.

### Material preparation

Ti-Ta-Hf-Zr ingot was fabricated by a cold-crucible levitation melting method using high purity sponge Ti and sponge Hf, Zr702 (commercial pure Zr), and high-purity Ta powder as raw materials, ingots of the alloys were re-melted five times in order to guarantee chemical homogeneity. The nominal compositions of the Ti-Ta-Hf-Zr alloys were Ti-37Ta-26Hf-13Zr, Ti-40Ta-22Hf-11.7Zr, and Ti-45Ta-18.4Hf-10Zr (wt.%), which were hereafter denoted as TTHZ-1, TTHZ-2, and TTHZ-3, respectively. The chemical compositions of the ingot alloys were determined by wavelength dispersive X-ray fluorescence (WDXRF) spectrometer (S4 Pioneer, Bruker, Germany). The results of chemical analysis of the TTHZ alloys (in wt.% and at.%) are listed in Table 1S.

### Microstructural characterization

Microstructures of the TTHZ alloys were observed by optical microscopy (OM) (Leica DM2500). The samples for OM observation were ground, polished, and etched with Kroll solution (distilled water 100 ml, nitric acid 5 ml, hydrofluoric acid 3 ml), consecutively. The average grain size of the TTHZ alloys were measured using image analysis software Image Pro Plus 6.0. Phase constitutions of bulk samples were determined by X-ray diffractometry (XRD) using a Cu Kα radiation over the range 20° ≤ 2*θ* ≤ 90° with an accelerating voltage of 40 kV and a current of 250 mV at room temperature. The samples for transmission electron microscope (TEM) observation were cut through electrical discharge machining (EDM) and then mechanically ground to a thickness of approximately 80 μm. Following this stage, TEM foils were punched, electro-polished in a solution composed of 59% methanol, 35% *n*-butyl alcohol, and 6% perchloric acid using a Fischione Model 120 Twinjet. TEM observations were performed at an accelerating voltage of 200 kV (JEOLJEM 2000FX).

### Mechanical property testing

Micro-hardness was measured using a Vickers micro-indenter (Buehler Micomet 2100) by loading with a 200 g load for 15 s on the samples. Nine different measurements were taken, and the minimum and maximum values were discarded before averaging.

Cylindrical samples with dimensions of diameter 5 mm and 8 mm long were machined using EDM for compression tests. The compressive properties of the TTHZ alloy samples were evaluated using a Materials Testing Systems at a deformation rate of 0.5 mm/min at room temperature. At least five samples were tested for each group of samples and the property data were the averaged values.

Tensile samples with a gauge section of 8 mm × 2 mm × 1 mm were prepared by EDM. An Instron 5567 testing system, equipped with an advanced video extensometer, was used to record the stress-strain data until fracture with a cross-head speed of 0.5 mm/min at room temperature. The tensile strength, yield strength, Young’s modulus, and elastic strain of the studied alloys were analyzed. Experiments were carried out on five samples and the average value of the five measurements was considered. The fracture surfaces of the tensile samples were examined by field-emission scanning electron microscopy (FESEM, ZEISS SUPRA 40 VP).

### MTS assay

A MTS assay was used to determine the cytotoxicity of TTHZ alloys. Disc samples with dimensions of 8 mm in diameter and 2 mm thick were prepared by EDM and sterilized at 180 °C for 3 h in an autoclave. The cytocompatibilities of TTHZ alloys were assessed using SaOS2 osteoblast-like cells (Barwon Biomedical Research, Geelong Hospital, Victoria, Australia) based on International Standard ISO10993-5^60^. The SaOS2 cells were seeded onto the surface of the TTHZ alloy discs at a density of 5 × 10^3^ cells per well in a cell culture plate. For comparison, commercially pure Ti (grade 2 ASTM) plates were also assessed.

The cell morphology of the SaOS2 cells on the surfaces of the TTHZ discs was observed by using SEM and a confocal microscope (Leica SP5, Leica Microsystems, Germany). For SEM observation, the cultured disc samples were fixed in 3.9% glutaraldehyde for 12 h at room temperature. Following this, the SaOS2 cells were dehydrated through progressive washings in 60%, 70%, 80%, 90%, 95%, and 100% ethanol solutions for 10 min per step. Following this procedure, the samples were chemically dried using hexamethyldisilazane and coated with gold for SEM observation. For confocal microscopy, the cell-seeded discs were fixed in 2% paraformaldehyde and permeabilized with 0.2% (v/v) triton-X100 in phosphate-buffered saline (PBS; Sigma-Aldrich) at room temperature, for 10 min each. The samples were then incubated with 1% phalloidin and 4′-6-diamidino-2-phenylindole (DAPI) overnight at 4 °C. The samples were washed three times with PBS between each of the aforementioned steps. The stained samples were stored in PBS for posterior use, and confocal microscopy observations of these samples were conducted within one week of staining.

### Statistical analysis

The values belonging to mechanical properties and cell adhesion density were expressed as means ± standard deviation (SD). Each mechanical test (tensile and compression) was conducted five times. The values were expressed as means ± standard deviation (SD). One-way ANOVA (ρ < 0.05) was used for the analysis of cell adhesion density and mechanical property values (micro-hardness, compressive yield strength, tensile and yield strength, elongation at rupture, Young’s modulus). The difference was considered significant at ρ < 0.05.

## Additional Information

**How to cite this article**: Lin, J. *et al.* Novel Ti-Ta-Hf-Zr alloys with promising mechanical properties for prospective stent applications. *Sci. Rep.* **6**, 37901; doi: 10.1038/srep37901 (2016).

**Publisher's note:** Springer Nature remains neutral with regard to jurisdictional claims in published maps and institutional affiliations.

## Supplementary Material

Supplementary Information

## Figures and Tables

**Table 1:** Comparison of mechanical properties of TTHZ alloys with the metals that are used for manufacturing stents.

Alloy	Tensile Strength (MPa)	Yield Strength (MPa)	Young’s Modulus (GPa)	Elastic admissible Strain (%)	Density (g/cm^3^)	References
TTHZ-1	1000.7 ± 108.2	1000.7 ± 108.2	79.1 ± 2.5	1.32 ± 0.18	8.7	This study
TTHZ-2	1160.2 ± 28.3	1132.2 ± 29.4	71.7 ± 2.3	1.58 ± 0.05	8.6	This study
TTHZ-3	1172.8 ± 45.1	1114.3 ± 61.0	77.0 ± 3.2	1.50 ± 0.13	8.7	This study
316L SS (ASTM F138 and F319; annealed)	586.0	331.0	190.0	0.17	7.9	7, 8
Tantalum	207.0	138.0	185.0	0.07	16.6	7
Nitinol (Austenite)	895.0	195.0–690.0	83.0	0.23–0.83	6.7	7, 9
Co-Cr	951.0–1220.0	448.0–648.0	210.0	0.21–0.31	9.2	7, 8, 9

(TTHZ-1: Ti-37Ta-26Hf-13Zr, TTHZ-2: Ti-40Ta-22Hf-11.7Zr, TTHZ-3: Ti-45Ta-18.4Hf-10Zr).
