# Supplementary material for: Novel Ti-Ta-Hf-Zr alloys with promising mechanical properties for prospective stent applications
Source: Sci Rep. 2016 Nov 29;6:37901. doi: 10.1038/srep37901 (PMC5126583; doi:10.1038/srep37901)
Supplement: Supplementary Information [file srep37901-s1.doc]

**Supplementary Information**

**Novel Ti-Ta-Hf-Zr alloys with promising mechanical properties for prospective stent applications**

By Jixing Lin^1,2^, Sertan Ozan^3^, Yuncang Li^4^, Dehai Ping^5^, Xian Tong^2^, Guangyu Li^1*^ and Cuie Wen^4*^

**Captions of Supplementary Tables and Figures**

Table S1 Chemical composition (wt.% and at.%) of studied Ti-Ta-Hf-Zr alloys.

Figure S1 Optical micrographs of micro-Vickers indents presenting shear bands around the indents: (a) TTHZ-1 (Ti-37Ta-26Hf-13Zr), (b) TTHZ-2 (Ti-40Ta-22Hf-11.7Zr), and (c) TTHZ-3 (Ti-45Ta-18.4Hf-10Zr).

Figure S2 Images of TTHZ alloy samples after compression test: (a) TTHZ-1, top-view, (a’) TTHZ-1, side-view; (b) TTHZ-2, top-view, (b’) TTHZ-2, side-view; and (c) TTHZ-3, top-view, (c’) TTHZ-3, side-view.

Figure S3 Tensile stress-strain curves of as-cast TTHZ alloys: (a) TTHZ-1 (Ti-37Ta-26Hf-13Zr), (b) TTHZ-2 (Ti-40Ta-22Hf-11.7Zr), and (c) TTHZ-3 (Ti-45Ta-18.4Hf-10Zr).

Figure S4 Fracture surface of as-cast TTHZ alloys: (a) TTHZ-1 (Ti-37Ta-26Hf-13Zr), (b) TTHZ-2 (Ti-40Ta-22Hf-11.7Zr), and (c) TTHZ-3 (Ti-45Ta-18.4Hf-10Zr).

Figure S5 SaOS2 cell adhesion density of as cast TTHZ alloys and CP-Ti after cell culture for 7 d. (TTHZ-1: Ti-37Ta-26Hf-13Zr, TTHZ-2: Ti-40Ta-22Hf- 11.7Zr, TTHZ-3: Ti-45Ta-18.4Hf-10Zr.)

Figure S6 Morphology of SaOS2 cells attached on the surface of TTHZ-2 (Ti-40Ta-22Hf-11.7Zr) after cell culture for 24 h.

Figure S7 Location of TTHZ alloys on the
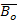
-
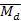
 map and the electronic parameters of TTHZ alloys (adapted and redrawn from [58]).

Table S1 Chemical composition of the studied Ti-Ta-Hf-Zr alloys (wt.% and at.%).

| Alloy name | Chemical composition | | | | | | |
| --- | --- | --- | --- | --- | --- | --- | --- |
|  | Ta | | Hf | | Zr | | Ti |
|  | wt.% | at.% | wt.% | at.% | wt.% | at.% |  |
| TTHZ-1 | 37.60 | 20.97 | 25.50 | 14.42 | 13.16 | 14.56 | Bal. |
| TTHZ-2 | 39.93 | 21.64 | 21.80 | 11.98 | 12.35 | 13.28 | Bal. |
| TTHZ-3 | 46.41 | 25.94 | 18.90 | 10.71 | 9.869 | 10.94 | Bal. |

**
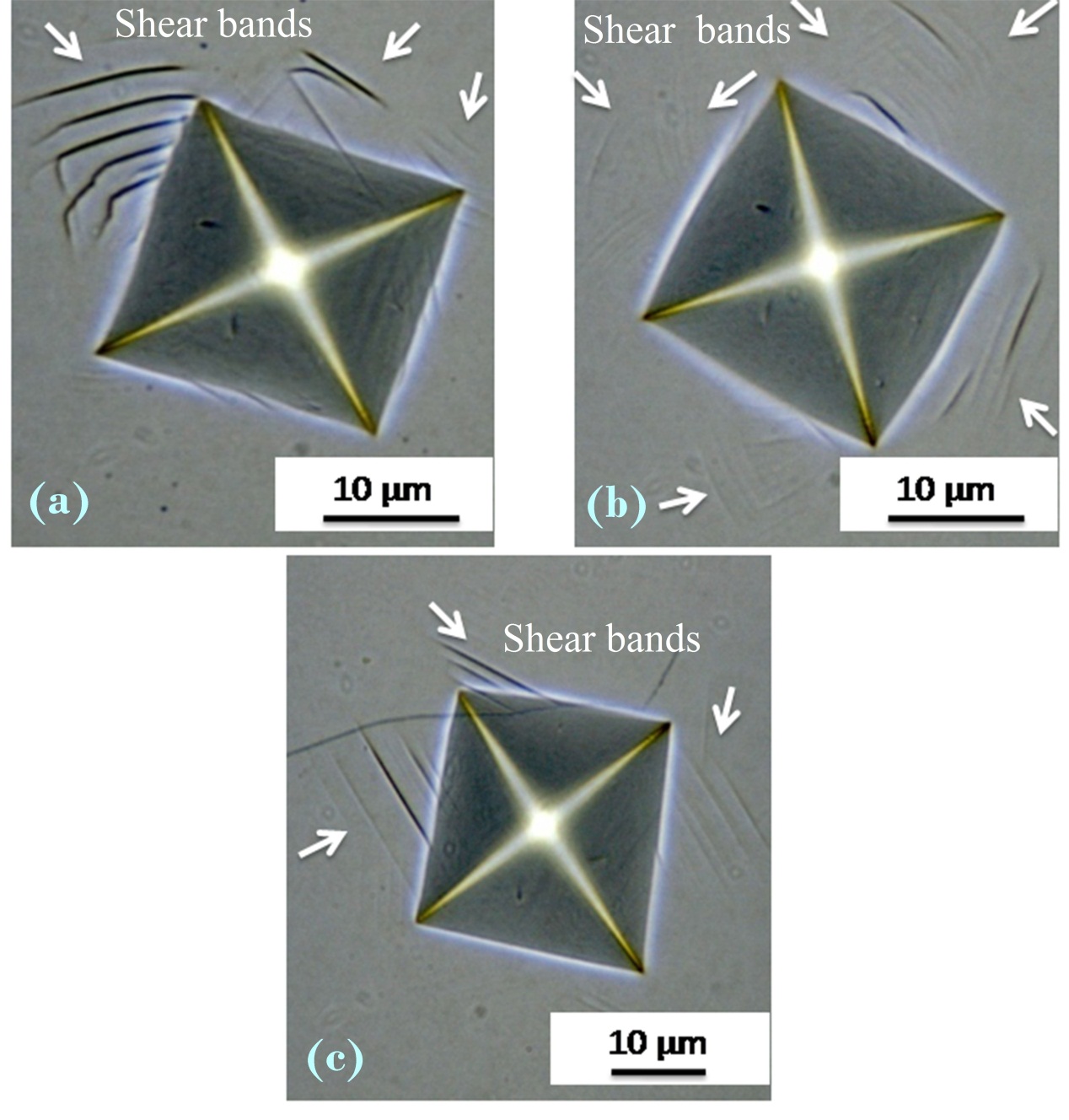
**

Figure S1 Optical micrographs of micro-Vickers indents presenting shear bands around the indents: (a) TTHZ-1 (Ti-37Ta-26Hf-13Zr), (b) TTHZ-2 (Ti-40Ta-22Hf-11.7Zr), and (c) TTHZ-3 (Ti-45Ta-18.4Hf-10Zr).


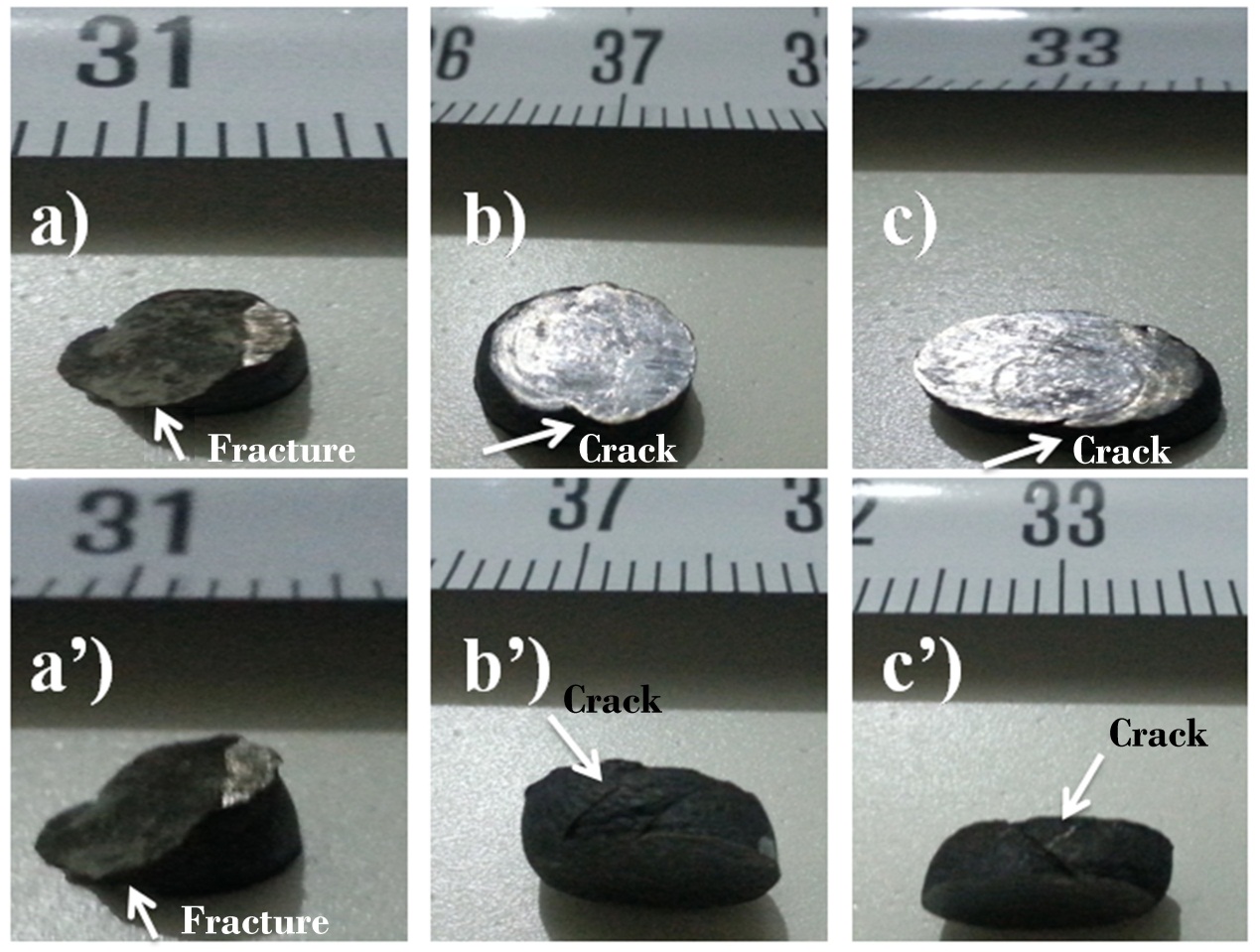


Figure S2 Images of TTHZ alloy samples after compression test: (a) TTHZ-1, top-view, (a’) TTHZ-1, side-view; (b) TTHZ-2, top-view, (b’) TTHZ-2, side-view; and (c) TTHZ-3, top-view, (c’) TTHZ-3, side-view.

**
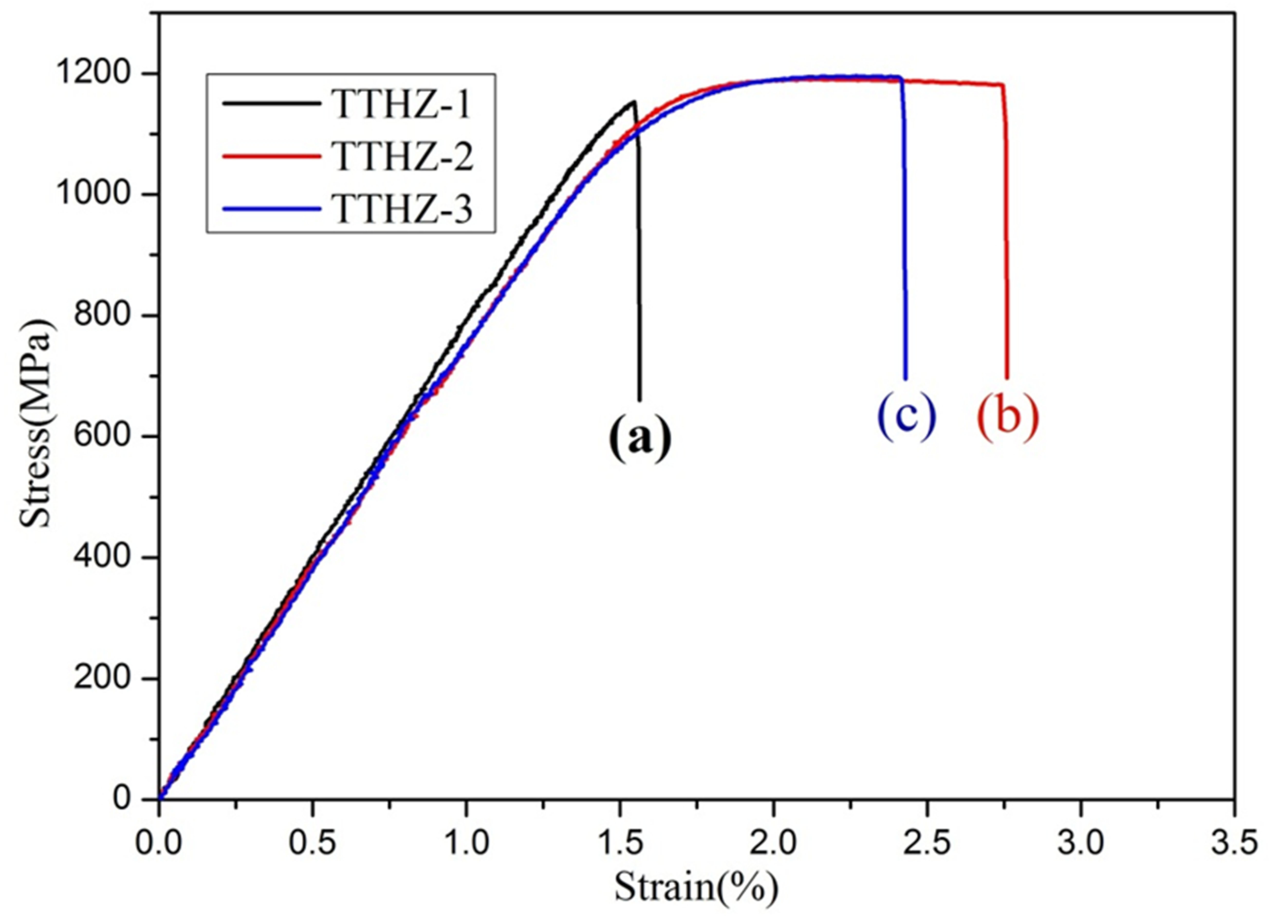
**

Figure S3 Tensile stress-strain curves of as-cast TTHZ alloys: (a) TTHZ-1: Ti-37Ta-26Hf-13Zr, (b) TTHZ-2: Ti-40Ta-22Hf-11.7Zr, and (c) TTHZ-3: Ti-45Ta-18.4Hf-10Zr.

**
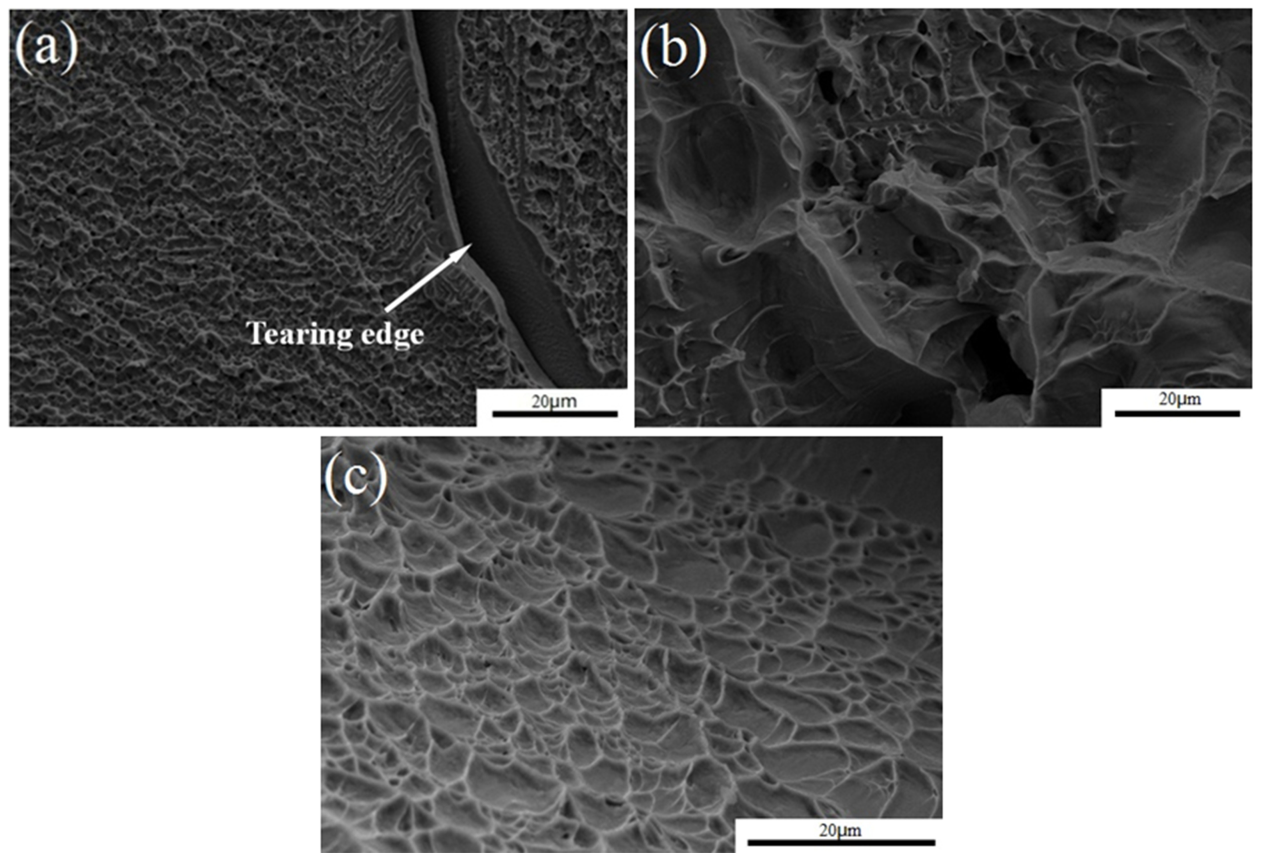
**

Figure S4 Fracture surface of as-cast TTHZ alloys: (a) TTHZ-1 (Ti-37Ta-26Hf-13Zr), (b) TTHZ-2 (Ti-40Ta-22Hf-11.7Zr), and (c) TTHZ-3 (Ti-45Ta-18.4Hf-10Zr).

**
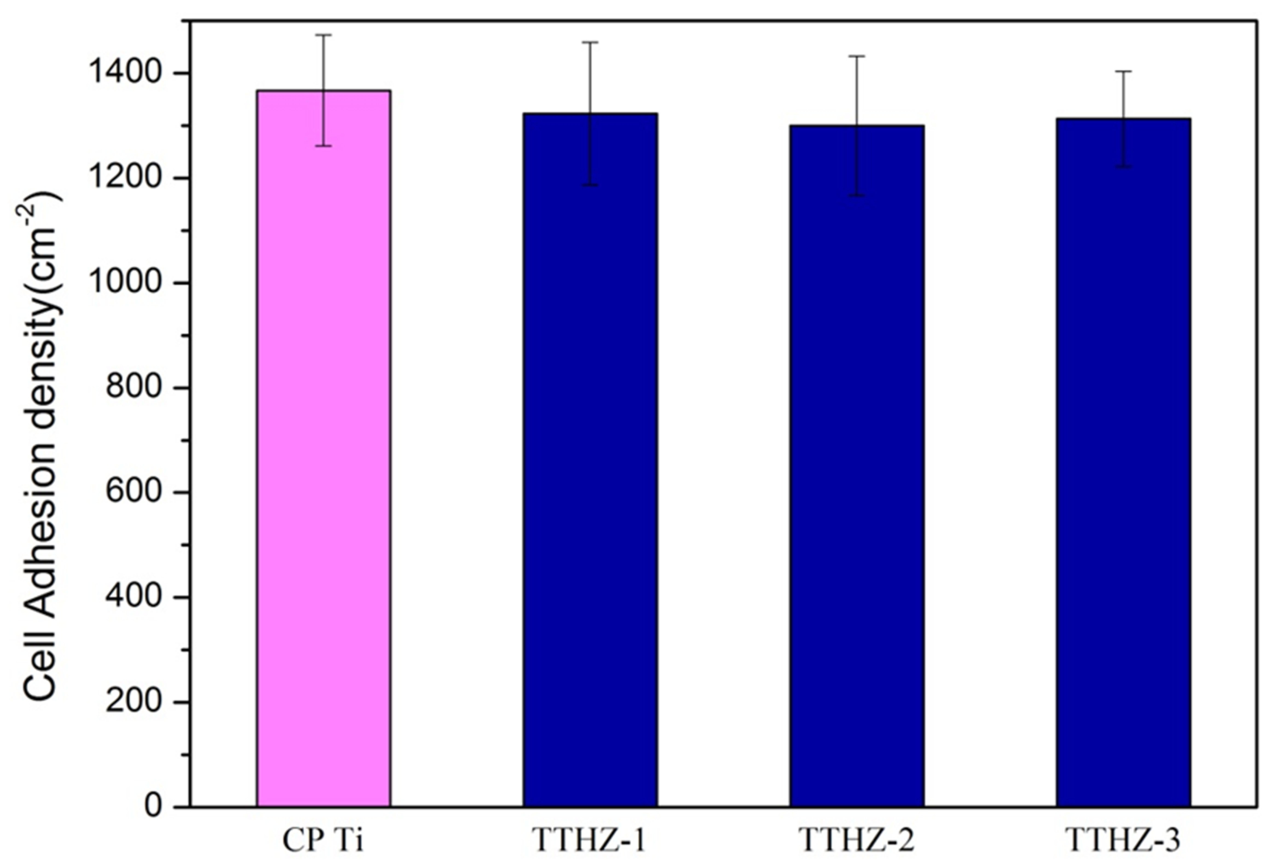
**

Figure S5 SaOS2 cell adhesion density of as cast TTHZ alloys and CP-Ti after cell culture for 7 d (TTHZ-1: Ti-37Ta-26Hf-13Zr, TTHZ-2: Ti-40Ta-22Hf- 11.7Zr, TTHZ-3: Ti-45Ta-18.4Hf-10Zr).

**
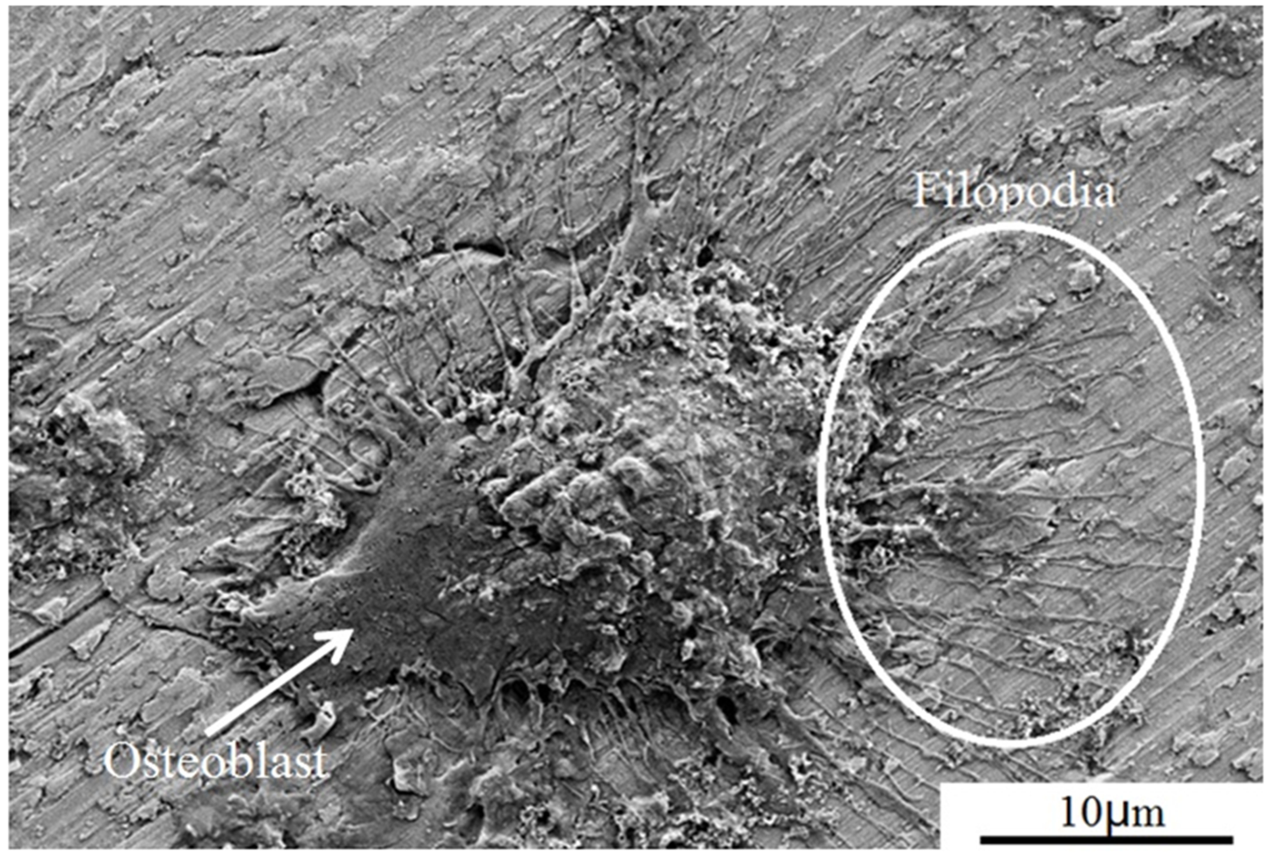
**

Figure S6 Morphology of SaOS2 cells attached on the surface of TTHZ-2 (Ti-40Ta-22Hf-11.7Zr) after cell culture for 24 h.

**
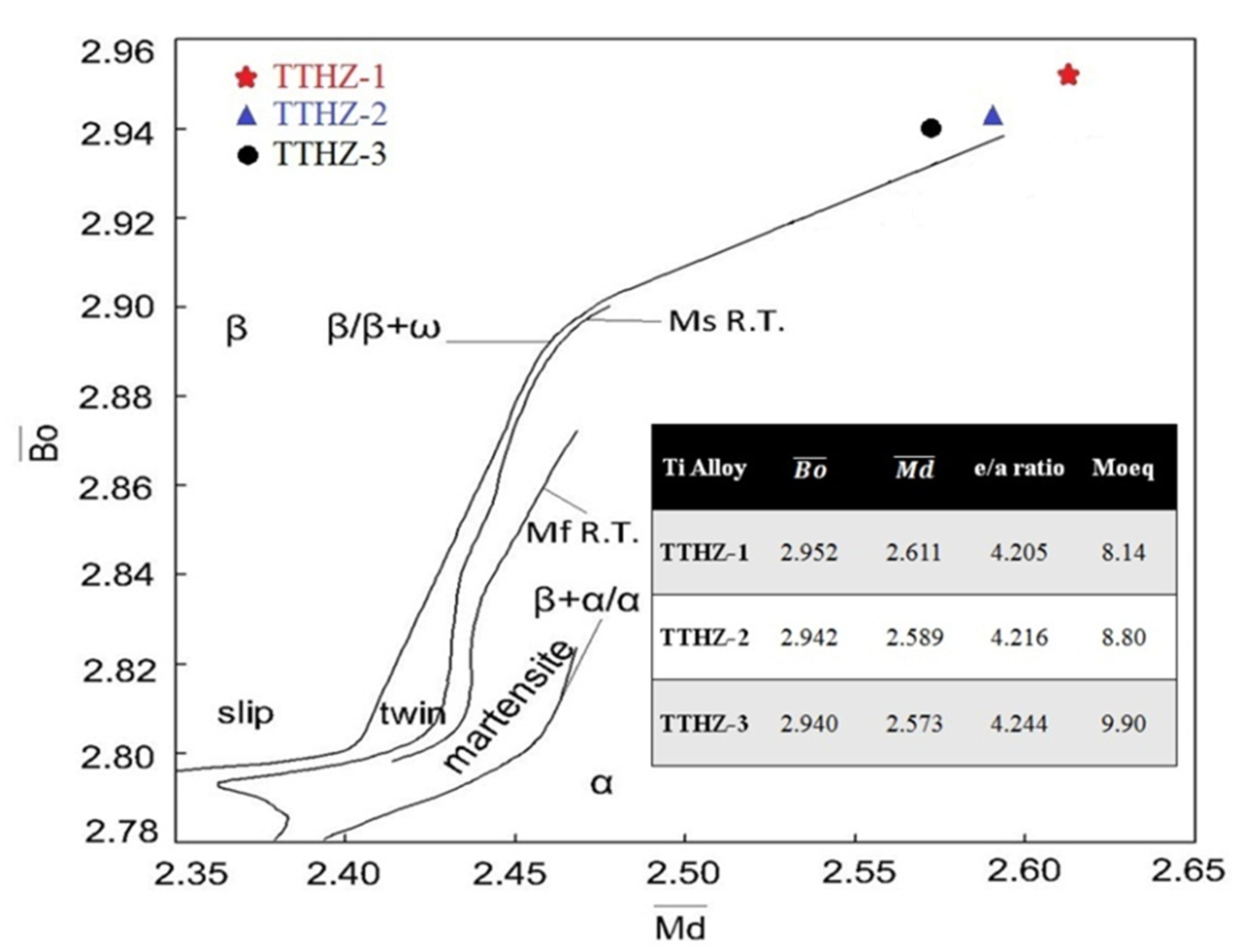
**

Figure S7 Location of TTHZ alloys on the
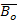
-
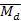
 map and the electronic parameters of TTHZ alloys (adapted and redrawn from [57]).
